# Effective NSAID treatment indicates that hyperprostaglandinism is affecting the clinical severity of childhood hypophosphatasia

**DOI:** 10.1186/1750-1172-1-24

**Published:** 2006-06-28

**Authors:** HJ Girschick, P Schneider, I Haubitz, O Hiort, H Collmann, M Beer, YS Shin, HW Seyberth

**Affiliations:** 1 Children's Hospital, University of Würzburg, Germany; 2 Clinic for Nuclear Medicine, University of Würzburg, Germany; 3 Children's Hospital, University of Lübeck, Germany; 4 Section of Pediatric Neurosurgery, University of Würzburg, Germany; 5 Dept. of Radiology, Section of Pediatric Radiology, University of Würzburg, Germany; 6 Children's Hospital, University of Munich, Germany; 7 Children's Hospital, University of Marburg, Germany

## Abstract

**Background:**

Hypophosphatasia (HP) is an inborn error of bone metabolism characterized by a genetic defect in the gene encoding the tissue-nonspecific alkaline phosphatase (TNSALP). There is a lack of knowledge as to how the variability and clinical severity of the HP phenotype (especially pain and walking impairment) are related to metabolic disturbances or impairments, subsequent to the molecular defect.

**Methods:**

We analyzed the changes in clinical symptoms and the prostaglandin (PG) metabolism in response to treatment with non-steroidal anti-inflammatory drugs (NSAIDs) in six children affected by childhood HP. In addition, by exposing HP fibroblasts to pyridoxal phosphate and/or calcium pyrophosphate *in vitro*, we analyzed whether the alterations in PG levels are sequelae related to the metabolic defect.

**Results:**

Childhood HP patients, who often complain about pain in the lower limbs without evident fractures, have systemic hyperprostaglandinism. Symptomatic anti-inflammatory treatment with NSAIDs significantly improved pain-associated physical impairment. Calcium pyrophosphate, but not pyridoxal phosphate, induced cyclooxygenase-2 (*COX-2*) gene expression and PG production in HP and normal fibroblasts *in vitro*.

**Conclusion:**

Clinical features of childhood HP related to pain in the lower legs may be, at least in part, sequelae related to elevated PG levels, secondary to the primary metabolic defect. Consequently, NSAID treatment does improve the clinical features of childhood HP.

## Background

Hypophosphatasia (HP) (MIM 241510) is an inborn error of bone metabolism, characterized by a genetic defect in the gene encoding the tissue-nonspecific alkaline phosphatase (TNSALP) [1-3]. The HP phenotype displays considerable clinical variability. Five major subtypes of HP (perinatal, infantile, childhood, adult and odontohypophosphatasia) have been described [4]. There are biochemical [5] and molecular data [6-9] indicating that the severity of the molecular genetic alterations and subsequent changes in the levels of TNSALP are major determinants of the clinical phenotype. However, there is a lack of knowledge about how these features and the severity of the phenotype are influenced by the molecular defect and/or subsequent metabolic disturbances or sequelae. This seems of particular importance since metabolic products accumulating in HP patients, like pyrophosphate [10,11], have the potential to provoke crystal-induced arthritis [12-16]. Since milder phenotypes, including the childhood form of HP, do not reduce the life span of the affected individuals significantly, there is considerably more time for such secondary metabolic phenomena to influence the HP phenotype. We have previously shown that childhood HP patients commonly complain about pain in the lower limbs, especially after physical exercise [17]. In our previous study, symptomatic treatment of HP patients with one course of non-steroidal anti-inflammatory drugs (NSAID) resulted in a significant clinical improvement in physical activity and a reduction in pain-related complaints [17]. So far, no long-term data on such a symptomatic treatment has been reported. As prostaglandins (PG) can induce appositional bone growth [18-20], we hypothesized that elevated PG values might be a reaction compensating for a mechanically incompetent bony structure, or it might be a sequel caused by the metabolic defect in HP [21]. In order to avoid blocking PG synthesis for a long period of time, we now have investigated the effect of repeated NSAID treatments, each lasting 8 weeks, with an interval of four weeks in between.

We hypothesized that the systemic elevation of PG levels seen in our patients was due to pyrophosphate stimulation of connective tissue cells including fibroblasts; PG synthesis by skin and synovial non-hypophosphatasia fibroblasts has already been established [14]. We, therefore, compared the prostaglandin synthesis occurring in normal as well as in HP fibroblasts exposed to pyridoxal phosphate and/or calcium pyrophosphate, two of the metabolic products that accumulate in hypophosphatasia. The goal of these analyses was to find out whether mechanisms secondary to TNSALP deficiency influence the clinical severity in patients diagnosed with childhood HP and whether pyrophosphate-stimulated HP fibroblasts are more prone to promote inflammation than healthy controls.

## Patients and methods

### Patients

The clinical features, biochemical data, genetic analysis and clinical effectiveness of a short-term NSAID treatment have been reported in detail previously, in this cohort of six patients [21]. In the present study, the same patient numbering system has been used. Genetic analysis has been reported previously: Patient #1 belongs to family C, patient #2 belongs to family E and patient #3 belongs to family A [9]. No genetic analysis is available for patients #4, #5 and #6. Six boys with childhood HP aged 2, 2.5, 3, 7.5, 9 and 14 years at initial presentation were followed up for 4.25, 3.5, 6.5, 4, 4, 3 years respectively, using a protocol which included patient's history, physical examination, radiographic imaging (skull, extremities, left carpus), abdominal ultrasound. Biochemical analysis of TNSALP metabolism in serum, plasma and urine is shown in table 1 and includes PG analysis in the urine. Growth, dental abnormalities, walking ability, kidney function and craniofacial complications were monitored. Bone mineral density (BMD) was measured initially and each year during follow-up. All patients had genua vara or valga and a waddling gait. Walking was delayed in all patients by periods ranging from 14 to 24 months (mean: 18 months). Gower's sign was present in five patients. The walking distance and quality of life was limited by diffuse pain of the lower legs in all patients. Craniosynostosis was present in three patients; two patients required a total of two or three neurosurgical procedures at the cranial vault for release of the intracranial pressure. Premature loss and loosening of deciduous teeth, affecting mainly the incisors, was noted in all patients. We have previously reported the measurement of BMD and markers of bone metabolism from five kindred including six subjects with childhood HP [21] who have been further analyzed in this study.

**Table 1:** Biochemical analyses

**patient**	**normal range**	**#1**	**#2**	**#3**	**#4**	**#5**	**#6**
TNSALP in leukocytes	2–18 nmol/min/mg protein	0.1	0	0.11	0.1	0.1	0.5
PEA in serum/plasma	0–60 μmol/l	25	Nd	98	6	7	97
PEA/Cr in urine	9–25 mmol/mol Cr	252	148	250	157	117	155
PLP in plasma	5–30 ng/ml	1039	408	185	324	446	165
**Age**	years	2	2,5	3	7,42	9	13

Abbreviations:

TNSALP tissue non-specific alkaline phosphatase,

PEA phosphoethanolamin,

Cr creatinine,

PLP pyridoxal phosphate

### Bone metabolic profile

The following assays were performed: serum calcium and phosphate, intact parathyroid hormone (PTH) (1–84 amino acids), TNSALP and total serum alkaline phosphatase (AP). In urine, deoxypyridinoline/creatinine ratio, hydroxyproline/creatinine ratio, calcium/creatinine ratio and phosphate excretion were measured. Calcium concentrations were measured by flame atomic absorption, phosphate and creatinine concentrations were measured by standard colorimetric assays. Intact PTH was measured by a 2-site immunoradiometric assay (Nichols Institute, Bad Nauheim, Germany). Analysis of TNSALP and its substrates was performed by Y. S. Shin, MD (Munich, Germany) [22]. Analysis of deoxypyridinoline/creatinine and hydroxyproline/creatinine levels in the urine was performed by K. Kruse, MD (†) and O. Hiort, MD (Lübeck, Germany) using enzyme immunoassays (Metra Biosystems Inc., Palo Alto, CA, USA) [23].

### Prostaglandin profile in the urine during NSAID treatment

The urinary PG profile was determined prior to and after 8 weeks of treatment with the NSAID naproxen (15 mg/kg per day in oral suspension). Treatment cycles were repeated according to clinical demands, *e.g*. when the patient's parents reported significant physical impairments due to pain. The effectiveness of the first treatment cycle in each patient has been reported previously [17]. Patients #1–6 received 2, 2, 7 (3 using naproxen, 4 using meloxicam), 3, 1 and 2 treatment cycles, respectively, each lasting 8 weeks. Patient #3 had developed pseudoporphyria after the third naproxen treatment cycle [24], therefore his treatment was switched to meloxicam (0.25 mg/kg oral suspension per day for 8 weeks). Meloxicam treatment was repeated four times in a prospective manner, with an 8-week pause in treatment in between, and was further evaluated for clinical response and laboratory changes (figures 2,3,4). A final analysis of the systemic index metabolite of prostaglandin E2 (PGE-M) was performed 8 weeks after the discontinuation of the last meloxicam cycle (treatment 5). Meloxicam oral suspension was obtained from Boehringer Ingelheim (Biberach, Germany). PGs and their metabolites were determined in 24-hour urine samples by capillary gas chromatography and mass spectrometry by H.W. Seyberth, MD (Marburg, Germany) [25], as previously reported [17]. Urinary excretion rate of PGE2 was used as an index of renal prostanoid activity. Renal PGE2 production did not change after treatment (paired t-Test, p = 0.1006). The excretion rate of 7**α**-hydroxy-5,11-diketotetranorprosta-1,16-dioic acid (systemic index metabolite reflecting systemic levels of PGE2, PGE-M) was used as an indicator of total body PG production [25].

**Figure 1:**
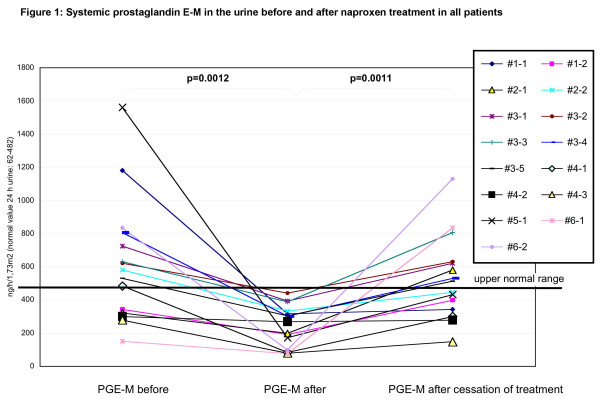
**Systemic prostaglandin E-M (PGE-M) in the urine before and after naproxen treatment in all patients**. The urinary PG profile was determined prior to, after 8 weeks of treatment and after another 8 weeks of cessation of treatment with the NSAID naproxen (15 mg/kg of an oral suspension per day). Treatment cycles were repeated according to clinical necessity, especially when the patient's parents reported significant physical impairment due to pain. The levels of PGs and their metabolites were determined in 24-hour urine samples by capillary gas chromatography and mass spectrometry. Systemic prostaglandin PGE-M production was evaluated in 15 treatment cycles (connected lines). Eight out of 15 revealed elevated levels above the normal range before the start of therapy. All PGE-M levels dropped into the normal range immediately after therapy (paired t-test, p = 0.0012). In addition, subsequent PGE-M measurements taken 8 weeks after treatment cessation showed a re-elevation in PGE-M levels (paired t-test, p = 0.0011).

**Figure 2:**
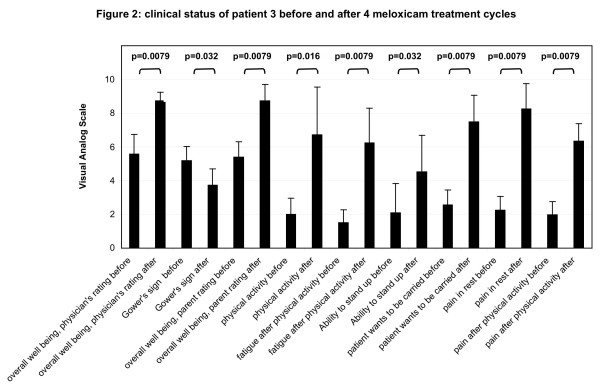
**Clinical status of patient 3 before and after four meloxicam treatment cycles**. Patient #3 was followed prospectively during four cycles of anti-inflammatory therapy using meloxicam. Each cycle lasted 8 weeks. The clinical status was evaluated with a parent's questionnaire and physicians rating using visual analogue scales ranging from 0 to 10 (p calculated by Mann Whitney's exact U-test).

**Figure 3:**
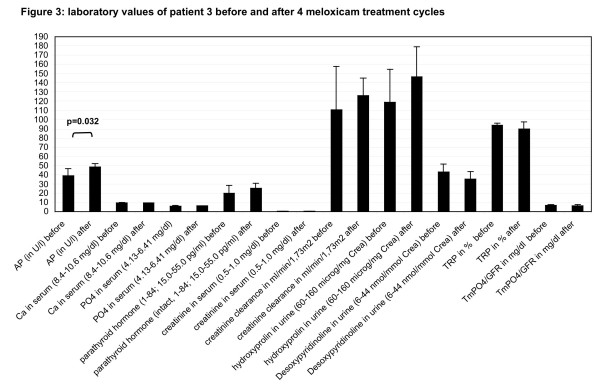
**Laboratory values of patient 3 before and after four meloxicam treatment cycles**. For figure legend see figure 2.

**Figure 4:**
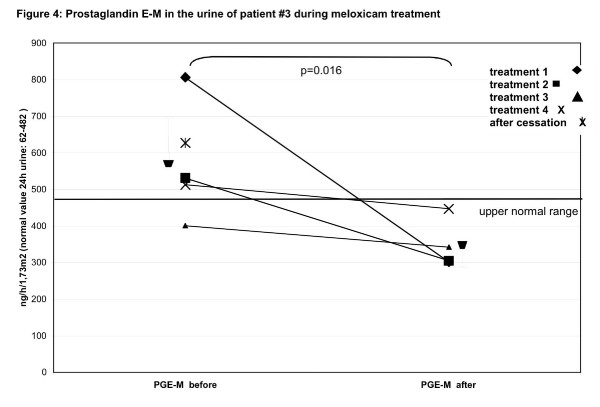
**Prostaglandin E-M (PGE-M) in the urine of patient #3 during meloxicam treatment**. The urinary PG profile was determined prior to and after 8 weeks of treatment with the NSAID meloxicam at 0.25 mg/kg oral suspension per day. Treatment cycles were repeated four times prospectively according to a predefined protocol. Therapy cycles were separated by an 8-week treatment pause. The levels of PGs and their metabolites were determined in 24-hour urine samples by capillary gas chromatography and mass spectrometry (p calculated by Mann Whitney's exact U-test). A final PGE-M analysis was done 8 weeks after discontinuation of the last meloxicam cycle (treatment 5, unconnected X). The  indicates the mean plus standard deviation of the different sample sets.

This study was funded by University funds only. The treatment was reviewed and approved by the ethics committee of the University of Würzburg, according to the principles of the Declaration of Helsinki.

### Cell culture

Human fibroblasts were prepared from normal human skin (control), from skin of a HP patient and from dura mater of another HP patient (both patients underwent neurosurgery for release of increased intracranial pressure; these patients were not included in the biochemical analyses shown below). Tissue samples were prepared by trypsin enzymatic digestion, maintained in culture following a method described previously [26]. The same amount of medium out of the same batch was used for the culture of the cells. Culture conditions between the samples were kept as comparable and constant as possible. The study was performed according to the principles of the Declaration of Helsinki. Isolation and culture of fibroblasts were approved by the ethics committee of the University of Würzburg, Germany. Fibroblasts were cultured in 10 cm^2 ^culture plates (Nunc) using RPMI-1640 medium (Invitrogen), supplemented with 10% fetal calf serum (FCS) (Biochrom KG, Berlin, Germany) at 37°C in a 5% CO_2 _humidified incubator. They were used between passages 10 and 12. For all experiments, fibroblasts (1 × 10^5^) were seeded in different culture plates and used at a confluent growth state. Normal and HP fibroblasts were stimulated with pyridoxal-5-phosphate (C_8_H_10_NO_6_P; Sigma Aldrich) at 0.5 micrograms/ml of culture medium for 48 hours *in vitro*. No pyridoxal-5-phosphate was present in the RPMI medium, however, pyridoxine hydrochloride was present at the same concentration both in the tested samples and controls. Stimulation with calcium pyrophosphate (Ca_2_P_2_O_7_; 200 microgram/ml culture medium, Aldrich) was performed for 48 hours *in vitro*. According the manufacturer's analysis, no calcium pyrophosphate was present in the RPMI culture medium. There was no available data on the content of both substances in the FCS supplement. Pyridoxal-5-phosphate (PLP) concentration was chosen according to the patients' blood levels. Calcium pyrophosphate (CPPD) concentration was chosen according to former publications [14,16]. The experiments were done independently and repeated four times each. The concentrations of CPPD and PLP in the supplemented medium were the same in the different test samples as the same batch of supplemented medium was used.

### Isolation of RNA and synthesis of first strand complementary DNA

Total cellular RNA was isolated from fibroblasts of each individual experimental set using the RNeasy mini kit (Qiagen) according to Qiagen's protocol. Approximately the same amount of cells were harvested from each sample. Contaminating genomic DNA was removed using RNase-free DNase (Qiagen). Aliquots of RNA from each experimental set were reversely transcribed into cDNA using Superscript 2 RNase H reverse transcriptase (RT) (Invitrogen). The cDNA content of the samples was adjusted by spectrophotometry. Two negative samples were prepared, one by adding only RT mix (RT+) without RNA and another lacking RT (RT-). Positive controls were run using total RNA isolated from tonsil tissue. The following reagents were added in each tube for the reverse transcription reaction mix: 8 **μ**l 5X buffer (Invitrogen), 4 **μ**l dTT (0.1 M) (Invitrogen), 2 **μ**l dNTP (Sigma), 2 **μ**l Superscript 2 RNase H-reverse transcriptase (Invitrogen) and 1 **μ**l Rnasin (Promega). The reaction mix along with RNA was initially incubated for 15 minutes at 70°C, followed by incubation at 42°C for 50 minutes and 70°C for 15 minutes, finally the tubes were kept on ice for 10 minutes. Tubes containing cDNA were stored at -70°C. Polymerase chain reaction (PCR) was performed to amplify cDNA of expressed genes of interest. The experiments were repeated four times each.

### Quantification of cDNA in samples

For quantification of cDNA in the different samples, PCR amplification for the housekeeping gene **β**-actin was performed in triplicate at 25, 30 and 32 cycles each, after the initial adjustment of the cDNA content was done by spectrophotometry. RT-PCR products were analyzed on 1.8% agarose gels. The gel image was acquired by using a gel documentation unit (Bio-Rad). The intensities of the PCR product bands were analyzed by "quantity one" software (Bio-Rad). The mean counts of the intensities of these triplets in the linear range of PCR amplification were calculated and were used to calculate the amount of cDNA (in **μ**l) of each experimental sample that contained the same relative content of housekeeping gene cDNA, as previously described [21,27,28]. The calculated volume of cDNA was subjected to further PCR amplification of expressed genes of interest. The amplified PCR product was again analyzed by agarose gel electrophoresis.

### Polymerase chain reaction (PCR)

The PCR was performed in a mastercycler (Eppendorf). The **β**-actin-adjusted amount of cDNA from the respective experimental sets was mixed with 20 **μ**l molecular biology grade water (Eppendorf), 2.5 **μ**l thermophilic DNA poly 10X buffer (Promega), 1.5**μ**l 25 mM MgCl_2 _(Promega), 0.5**μ**l 10 mM dNTP (Sigma) and 0.25 **μ**l of 50 pmol 3' and 5' primers (MWG, Ebersberg, Germany) in a total volume of 25 **μ**l. The primer pairs used in the experiment were as follows:

COX-1 sense 5' GGGCTGATGGTCTTAAATGC 3',

COX-1 antisense 5' GCCAGGAACACAACACTTTG 3',

COX-2 sense 5' CAGCACTTCACGCATCAGTT 3',

COX-2 antisense 5' CAGCAAACCGTAGATGCTCA3',

beta actin sense 5'GTCCTCTCCCAAGTCCACACA3',

beta actin antisense 5'CTGGTCTCAAGTCAGTGTACAGGTAA3'.

All cyclooxygenase (COXs) primer pairs were designed from the published human cDNA sequence data (NCBI Genbank) using primer design software (Primer 3', Whitehead Institute of Biomedical Research, USA). The PCR of the target genes was done at the following settings: 5 minutes initial denaturation at 95°C in the first cycle, followed by 1 minute denaturation at 94°C, 1 minute annealing at 60°C and 1 minute elongation at 72°C for all the primer sets. We used 38 cycles for each experiment to see the expression of our target sequences, as described previously [27,28]. Experiments were repeated four times each. Intensities of the PCR product bands were calculated in a way that allowed comparison of different experiments. PCR band intensities of individual experiments (pyrophosphate and/or pyridoxal phosphate exposed and controls) were added and normalized to 100%. The percentages of the relative gene expression at a given time point in the four sets of experiments were added up, and the mean of relative cDNAexpression was calculated, as previously described [27,28].

### Enzyme linked immunosorbant assay (ELISA)

PGE2 concentrations in the supernatants of exposed and control fibroblast cultures were assessed using a high sensitivity PGE2 enzyme immunoassay kit (Assay Designs, Ann Arbor, MI, USA). The kit uses a monoclonal antibody coupled to alkaline phosphate, which binds PGE2 in a given sample. ELISA plates were incubated at 4°C for 24 hours after addition of samples, antibody and other required chemicals in each well. Excess reagents were washed away and substrate was added, followed by incubation at 37°C for 1 hour. The yellow color generated in the wells after addition of a stopping solution was analyzed using a microtiter plate reader at 405 nm. The intensity of the bound yellow color was inversely proportional to the concentration of PGE2 in the standards and samples. The measured optical density was used to calculate the concentration of PGE2, which is shown as the mean of quadruple experiments in figure 6.

**Figure 5:**
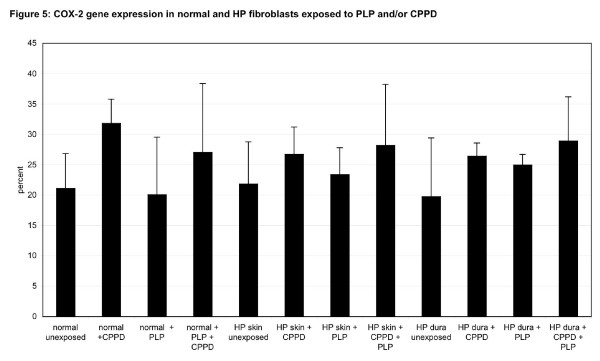
***COX-2 *gene expression in normal and HP fibroblasts exposed to pyridoxal phosphate (PLP) and/or calcium pyrophosphate (CPPD)**. Semiquantitative PCR was performed to analyze the expression of the *COX 2 *gene in normal and HP fibroblasts isolated from the skin and dura mater after exposure to PLP and/or CPPD *in vitro*. In order to allow comparisons, the expression was normalized to a 100% in each of the three groups of fibroblasts. Statistical analysis was done by 3-way ANOVA.

**Figure 6:**
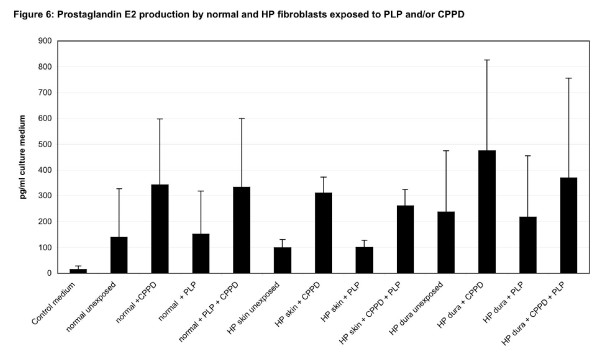
**Prostaglandin E2 (PGE2) production by normal and HP fibroblasts exposed to pyridoxal phosphate (PLP) and/or calcium pyrophosphate (CPPD) *in vitro***. PGE2 concentrations in the culture supernatant of exposed and control fibroblast cultures were assayed using a high sensitivity PGE2 enzyme.

### Statistical analysis

Mann Whitney's exact U-test was used for analyzing the changes in laboratory values (including PG data) and clinical status of patient #3 before and after treatment with meloxicam. Student's t-test was performed for comparison of the PG levels before and after treatment of all patients [17]. Three-factorial analysis of variance (3-way ANOVA) was performed to describe differences in the *COX-1, COX-2 *gene expression, in addition to PG production of fibroblasts *in vitro*. P values smaller than 0.05 were considered significant, indicating a difference between the initial values and the follow-up data. Statistical analysis was performed by Dr. I. Haubitz.

## Results

### Bone metabolism in patients

The diagnosis of childhood HP was based on clinical signs and confirmed in all patients by reduced serum alkaline phosphatase (AP) activity and elevated AP substrate, including phosphoethanolamine (PEA), levels in urine and elevated pyridoxal-5'-phosphate (PLP) in plasma [21]. TNSALP, measured in leukocytes, was at the lowest detectable level in all patients (mean 0.15 nmol/min/mg protein, range 0–0.5 nmol/min/mg protein, normal range 2–18 nmol/min/mg protein) (Table 1). Genetic analysis of the *TNSALP *gene has been previously reported [9]. Serum calcium concentrations were normal and phosphate levels were in the upper normal range. Interestingly, markers of bone turnover, including collagen crosslinks and hydroxyproline/creatinine levels in the urine, were unremarkable in all patients. The results obtained for patient #3 during the long-term prospective NSAID treatment with meloxicam are summarized in figures 2,3,4.

### Evaluation of "on demand" NSAID treatment in all patients

Previously, we have shown that one single 5-week long NSAID treatment cycle reduced PG levels in the patients studied [17]. During the follow-up, the anti-inflammatory treatment with naproxen was reinitiated in response to demand from the parents and patients because of clinical deterioration. Over periods of 4.25, 3.5, 6.5, 4, 4 and 3 years, 2, 2, 7, 3, 1 and 2 treatments, respectively, were demanded by the patients. Prior to NSAID treatment, the urinary PGE2 levels, reflecting renal PG production, were essentially within the normal range. No change was noted after 8 weeks of NSAID treatment (data not shown, paired 16 t-test, p = 0.1006). When systemic prostaglandin PGE-M production was evaluated (in 9 out of 15 treatment cycles) elevated levels (above the normal range) were detected before the therapy was started (figure 1). All PGE-M levels dropped into the normal range immediately after therapy (paired t-test, p = 0.0012) and did rise again after discontinuation of treatment (paired t-test, p = 0.0011) (Figure 1).

### Long-term prospective evaluation of NSAID treatment

Based on our previous study of NSAID treatment [17], we wanted to prospectively evaluate NSAID treatment in order to analyze its effects on the clinical status, bone metabolism and bone mineral density. As indicated in material and methods, patient #3 was followed during four cycles of meloxicam treatment over one year. Meloxicam was selected after the patient developed pseudoporphyria after three naproxen treatments. Each treatment cycle lasted 8 weeks and was followed by a pause of 8 weeks.

The clinical status of this patient (#3) was evaluated using a questionnaire, which has been evaluated and used previously [17]. The parents of the patient were asked to estimate overall well-being, physical activity, fatigue, ability to stand up, aversion to walking, pain in rest and pain after physical activity, before and after 8 weeks of treatment for each treatment cycle (figure 2) (table 2). In all these categories a statistically significant improvement was noted during each treatment cycle (p indicated in figure 2, Mann Whitney's exact U-Test). After each treatment pause of 8 weeks, however, the patient's physical status had worsened again. Each treatment cycle seemed to be of comparable effectiveness. The overall well-being was evaluated by a visual analogue scale from 0 – 10, with 0 indicating extremely reduced and 10 indicating normal. Physical activity was evaluated on a scale from 0 – 10, 0 indicating less active than normal kids, 5 indicating equal activity and 10 indicating a higher activity. Fatigue after physical activity was evaluated on a scale from 0 (rapid fatigue) to 5 (normal fatigue) to 10 (less fatigue) compared to normal kids after physical activity. The ability to stand up without the help of the arms was evaluated using 0 (not possible), 5 (significantly reduced) and 10 (not impaired). The wish to be carried was evaluated with 0 indicating a lot of instances to 10 indicating never. Whether pain was present during rest was evaluated from 0 (every night and day, very often) to 10 (once in a while, normal frequency). Pain after physical activity was evaluated from 0 (always) to 10 (never). In addition to the parents rating, the overall well-being was also judged by a "blinded" physician, using the same visual analogue scale as the parents. The presence of a Gower's sign was rated from 0 (not present) to 10 (bad, severe muscle weakness of the lower extremities). In similarity to the parent's rating, the physician's rating of these two manifestations also showed a significant improvement of the physical status of patient #3 during each treatment cycle (Mann Whitney's exact U-Test: p = 0.0079, p = 0.032; respectively) (figure 2).

**Table 2:** Clinical evaluation of patient #3 using meloxicam as anti-inflammatory treatment

	1. Cycle		2. Cycle		3. Cycle		4. Cycle		after 8 wks
time	before	after	before	after	before	after	before	after	pause

overall well being, **physician's global rating**	6	9	6	9	5	9	7	8	4
Gower's sign	4	3	5	4	5	3	6	5	6
overall well being, **parents' global rating**	5	10	5	8	5	9	7	8	5
physical activity	2,2	9,6	3,5	7	1,2	7,5	1,2	2,9	2
fatigue after physical activity	0,5	9,3	1,5	5	2,6	5,4	1,5	5,3	1,5
Gower's sign	2,3	6,4	5	6,4	0,8	2,5	1,2	2,9	1,3
patient wants to be carried	1,2	9,3	3,5	5,5	2,9	7,6	2,5	7,6	2,8
pain in rest	3	7	1,8	7	3,25	9,6	1,6	9,5	1,7
pain after physical activity	1,7	7	3,3	5	1,6	7,3	1,9	6,1	1,5

Laboratory analysis was done before and immediately after treatment, including a variety of parameters of bone metabolism (figure 3). There was no significant change in the concentration of calcium, phosphate, parathyroid hormone and creatinine in the serum, measured before and after treatment. Urinary excretion of hydroxyproline and deoxypyridinoline did not differ significantly before and after treatments. Functional analysis of the kidney including creatinine clearance, phosphate reabsorption (TRP) and phosphate transport maximum (TmPO4) were unchanged. All these values were within the normal range. However, serum alkaline phosphatase, measured before and after treatment, showed a significant increase from a mean of 39 U/l to 48.5 U/l (p = 0.032, Mann Whitney's exact U-Test) (figure 3). A selective analysis of TNSALP over time was not performed.

We also evaluated PG concentrations in the urine of patient #3 as indicated in figure 4. A significant reduction of systemic PGE-M levels, which were elevated in four out of five pre-treatment periods, was noted (figure 4) (Mann Whitney's exact U-Test: p = 0.016). After treatment, the PGE-M concentration was in the normal range. Renal PGE2production, which was not elevated before treatment, did not change significantly during the treatment cycles (Mann Whitney's exact U-Test: p = 0.3).

### *In vitro *cyclooxygenase gene expression in normal and HP fibroblasts exposed to pyridoxal phosphate (PLP) and calcium pyrophosphate (CPPD)

In order to evaluate a potential stimulation of normal and HP fibroblasts with PLP and/or CPPD *in vitro*, *COX-1 *and *COX-2 *gene expression was analyzed in these cells. PGE2 production was assessed by measuring PGE2 concentration in the supernatant, using the ELISA technique. *COX-1 *gene expression did not show a consistent change when CPPD was added either to normal or to HP skin fibroblasts, or HP fibroblasts isolated from the dura mater (3-way ANOVA, p = 0.16). In addition, no significant change was noted when PLP was added to the cultures, compared to the non-treated cells in each group (3-way ANOVA, p = 0.93). When PLP together with CPPD were added, a slight statistically insignificant increase in gene expression was noted in normal and HP skin fibroblasts. However, a reduction in the gene expression was found in HP dura mater fibroblasts. None of these changes in *COX-1 *expression, however, were statistically significant.

In normal fibroblasts, the addition of PLP did not alter *COX-2 *gene expression, whereas in the two HP fibroblast types, a slight increase of *COX-2 *production was noted (3-way ANOVA, p = 0.71) (figure 5). When CPPD was added to previously non-treated normal or HP fibroblasts, a significant increase of *COX-2 *gene expression was noted in each fibroblast type (3-way ANOVA, p = 0.0067). When PLP together with CPPD was added, an increase in *COX-2 *expression was noted in normal and HP skin fibroblasts, which was comparable to the stimulation with CPPD alone (figure 5). When these latter two sets were compared, no significant difference was noted (3-way ANOVA, p = 0.62), indicating that PLP did not contribute significantly to the *COX-2 *gene induction and PG production.

### *In vitro *prostaglandin production by normal and HP fibroblasts exposed to pyridoxal phosphate (PLP) and calcium pyrophosphate (CPPD) crystals

When PG concentrations were measured in the culture supernatant, a significant increase of PGE2 production was noted after calcium pyrophosphate was added to the cultures of either normal fibroblasts, HP skin or HP dura mater fibroblasts (3-way ANOVA, p = 0.011). No effect was observed when PLP was added to the cultures alone (3-way ANOVA, p = 0.88). No synergistic effect was noted when CPPD was added together with PLP (3-way ANOVA, p = 0.9) (figure 6).

## Discussion

Each of the childhood HP patients experienced a significant improvement of their physical status when treated with NSAIDs. This clinical effect was striking and could be noted in a "treatment on demand" setting involving all patients, as well as in repetitive treatments involving one patient. The latter was treated prospectively with the anti-inflammatory medication meloxicam for one year, including treatment pauses. Treated patients reported that the positive effect of the therapy was already noticeable within a few days after starting the NSAID treatment. After drug withdrawal, the positive effect on physical activity persisted for 3–6 weeks. Patient #3 did not experience an effect lasting longer than 3–4 weeks after NSAID therapy. This was consistent with a rise in PG levels before each treatment cycle. An excessive rebound of PG synthesis after cessation of COX blockade was not noted. An extensive concomitant laboratory analysis did not reveal changes in bone metabolism, kidney function and parathyroid function related to NSAID treatment. However, a significant increase of alkaline phosphatase activity in the serum was noted. So far, it is not clear whether this means an actual increase in TNSALP function or concentration, or whether it is caused by a variation in sample results measured at different time points. Nevertheless, it is important to analyze this particular effect in more detail in the future, because induction of *TNSALP *gene expression might be a therapeutic target in HP.

The pattern in mineralization obtained over the whole period of four years through bone mineral density measurements in the patient 3 undergoing repetitive treatments did not differ from that obtained from patients treated on an "on demand" basis for a limited time only. The improvements in the physical activity noted by the parents of this child during the treatment cycles, however, did not seem to influence the dynamics of bone mineral density to a noticeable extent during the follow-up of 4 years. Initially, we had feared that a PG synthesis blockade could even worsen bone mineralization, since PGs may have an inhibitory effect on bone formation rather than a positive effect. One can only speculate whether a long-term continuous anti-inflammatory and analgesic therapy can lead to an improvement or, at least, to a stabilization of the bony structure, secondary to an improvement of the physical activity.

*In vitro *analysis showed that normal and HP fibroblasts are equally sensitive to calcium pyrophosphate exposure. A significant increase of *COX-2 *gene expression and subsequent increase of PGE2 concentrations in the supernatant were noted in fibroblasts isolated from normal skin, HP skin and HP dura mater, after addition of calcium pyrophosphate but not pyridoxal phosphate. There was no HP-specific effect, showing that HP fibroblasts are particularly oversensitive to the exposure of calcium pyrophosphate. Thus, this study provides evidence that the elevation of PGs in HP might be based on the impaired clearance of calcium pyrophosphate resulting from alkaline phosphatase deficiency. Our data suggest that PGs can reduce the physical activity of childhood HP patients [17]. Physical inactivity could lead to a further impairment of bone mineralization, especially in the background of this genetic defect. Strenuous physical work carried out by patient #6 might have contributed to an increase of bone formation, which reached physiological levels. In contrast, patient #3, who was treated with NSAID for a total duration of 32 weeks, showed no improvement in bone mineral density. Thus, physical activity seems to have a more positive impact on mineralization in HP than normalization of potentially elevated PGs alone. Further studies are needed to understand the secondary metabolic phenomena associated with this inborn defect of bone metabolism and affecting the severity of the clinical phenotype.

## Abbreviations

AP – alkaline phosphatase

COX – cyclooxygenase

CPPD – calcium pyrophosphate

HP – hypophosphatasia

NSAID – non-steroidal anti-inflammatory drug

PCR – polymerase chain reaction

PEA – phosphoethanolamine

PG – prostaglandins

PGE2 – prostaglandin E2

PGE-M – systemic index metabolite of prostaglandin E2

PLP – pyridoxal phosphate

TmPO4 – tubular transport maximum of phosphate

TNSALP – tissue non-specific alkaline phosphatase

TRP – tubular absorption of phosphate

## Competing interests

The author(s) declare that they have no competing interests.

## Authors' contributions

HJH has written the manuscript, performed the clinical work and was in charge of patient care. PS performed the bone mineral density measurements plus data analysis, HC analyzed data and contributed significantly to the writing of the manuscript in addition to clinical care. IH performed statistical analysis and has contributed substantially to the manuscript. MB was responsible for the radiologic analysis. OH and YSS performed bone metabolism analyses, HWS performed the prostaglandin measurements. All authors read and approved the final manuscript.
